# Tx/Rx Head Coil Induces Less RF Transmit-Related Heating than Body Coil in Conductive Metallic Objects Outside the Active Area of the Head Coil

**DOI:** 10.3389/fnins.2017.00015

**Published:** 2017-01-26

**Authors:** Zoltan Nagy, Aaron Oliver-Taylor, Andre Kuehne, Sigrun Goluch, Nikolaus Weiskopf

**Affiliations:** ^1^Laboratory for Social and Neural Systems Research, University of ZürichZürich, Switzerland; ^2^Wellcome Trust Centre for Neuroimaging, University College LondonLondon, UK; ^3^Department of Neonatology, Institute for Women's Health, University College LondonLondon, UK; ^4^Center for Medical Physics and Biomedical Engineering, Medical University of ViennaVienna, Austria; ^5^MR Center of Excellence, Medical University of ViennaVienna, Austria; ^6^Department of Neurophysics, Max Planck Institute for Human Cognitive and Brain SciencesLeipzig, Germany

**Keywords:** implant, RF heating, safety, FDTD simulation, ASTM, neuroimaging

## Abstract

The transmit–receive (Tx/Rx) birdcage head coil is often used for excitation instead of the body coil because of the presumably lower risk of heating in and around conductive implants. However, this common practice has not been systematically tested. To investigate whether the Tx/Rx birdcage head coil produces less heating than the body coil when scanning individuals with implants, we used a 3T clinical scanner and made temperature measurements around a straight 15 cm conductor using either the Tx/Rx body or the head coil for excitation. Additionally, the transmitted fields of a Tx/Rx head coil were measured both in air and in gel using a resonant and a non-resonant **B** field probes as well as a non-resonant **E** field probe. Simulations using a finite-difference time domain solver were compared with the experimental findings. When the body coil was used for excitation, we observed heating around the 15 cm wire at various anatomical locations (both within and outside of the active volume of the head coil). Outside its active area, no such heating was observed while using the Tx/Rx head coil for excitation. The **E** and **B** fields of the Tx/Rx birdcage head coil extended well-beyond the physical dimensions of the coil. In air, the fields were monotonically decreasing, while in gel they were more complex with local maxima at the end of the ASTM phantom. These experimental findings were line with the simulations. While caution must always be exercised when scanning individuals with metallic implants, these findings support the use of the Tx/Rx birdcage head coil in place of the body coil at 3T in order to reduce the risk of heating in and around conductive implants that are remote from the head coil.

## Introduction

The radio frequency (RF) transmit field of an MRI scanner can induce currents on implanted conducting structures. These currents can lead to potentially harmful localized tissue heating. During an MRI examination, heating in conductors is induced by the oscillating local electric field. The fact that conductors with sharp edges concentrate the electric field exacerbates the issue (Purcell and Morin, 2013). The highest risk is posed by loops of conductors (Dempsey et al., 2001) or the tips of elongated metallic wires that are insulated along their length except at the ends (Yeung et al., 2002; Nyenhuis et al., 2005; Mattei et al., 2008). The local electrical field strength along the implant direction has been shown to strongly affect RF heating (Nordbeck et al., 2008). Furthermore, the amount of heating depends on the length and diameter (Armenean et al., 2004b) as well as the resistivity of the wire (Armenean et al., 2004a). Depending on the experimental setup, temperature rises of 30°C and above have been measured *ex vivo* (Konings et al., 2000; Smith et al., 2000; Nitz et al., 2001). *In vivo*, when the focal point of heating is perfused and thus naturally cooled, the measured temperature increases are usually lower but can still cause tissue damage. In muscle tissue, which is less well-perfused and hence has a reduced thermal conductivity, heating has been found to match the extent of findings from phantom experiments (Luechinger et al., 2005). However, all the above studies made use of large body coils for RF transmission. This paper investigates whether the use of a transmit–receive (Tx/Rx) birdcage head coil lowers the risk for heating around metallic implants when those implants reside in an area of the body that is entirely outside the volume of the coil. For such cases the Tx/Rx coil is often recommended (see for example Carmichael et al., 2007; Sankar and Lozano, 2011; Zrinzo et al., 2011) instead of the body coil. Albeit reasonable, this claim has not been tested systematically with implants that are entirely outside the volume of the Tx/Rx coil.

During an examination in a 1.0 T MRI scanner, an individual suffered a lesion adjacent to the tip of the implanted deep brain stimulation electrodes (Henderson et al., 2005). In this case, the examination was performed using the Tx/Rx body coil at 1.0 T instead of the Tx/Rx head coil at 1.5 T. The manufacturer of the implant specifically recommended the latter. It is important to note that the Tx/Rx birdcage head coil should not be universally assumed to be safe (Rezai et al., 2004) just because its use was deemed safe for a particular metallic prosthesis (Benbadis et al., 2001) or because at one field strength it has been shown to carry less risk than the body coil (Nyenhuis et al., 1997). In another case, even though the Tx/Rx birdcage head coil was deemed safe at 1.5 T, its use at 1.0 T led to the injury of an elderly patient (Spiegel et al., 2003). In both of these cases at least part of the implant was located in an area of the body that was within the physical volume of the Tx/Rx head coil. What happens when the implant is entirely outside the physical volume of the Tx/Rx head coil remains unclear.

Many implants have been studied systematically (Shellock, 2013). However, due to the sheer number of these devices it is not possible to test every single one. In addition, volunteers of research studies and patients undergoing clinical MRI examinations often do not know which implant they have. Even if they knew the exact type, make, and part number of their implant, manufacturers reserve the right to change the composition of the implant without changing the part number (Shellock and Kanal, 1996).

The American College of Radiology pointed out in their recommendations for safe MRI practice that “decisions based on published MR safety and compatibility claims should recognize that all (…) claims [of MR safety and compatibility] apply only to the specifically tested conditions….” (Kanal et al., 2013). In general, phantom experiments for MR heating tests cannot establish an unequivocal worst-case scenario for the entire patient or volunteer population (Kainz, 2007). The amount of heating depends not only on the known properties of the implant but also on its immediate surroundings (Konings et al., 2000), which are frequently unknown for *in vivo* scenarios. Under certain conditions, which depend on the length of wire, loop diameter, and inductive/capacitive coupling with external objects, an implant may become resonant at the transmit frequency. When resonant, power absorption of the implant increases significantly. Hence, predicting the extent of heating around an unknown implant due to the RF field is challenging.

Rather than establishing a worst-case scenario for a particular implant, the aim of the current study is to compare the extent of heating around a conductive metal wire while using either the Tx/Rx body or head coils. Both MR experiments and simulation comparisons were performed. In particular, these tests assess whether it is reasonable to follow the common practice of using the Tx/Rx head coil in place of the Tx/Rx body coil to reduce the risk of heating around conductive implants.

## Materials and methods

**Figure 1:**
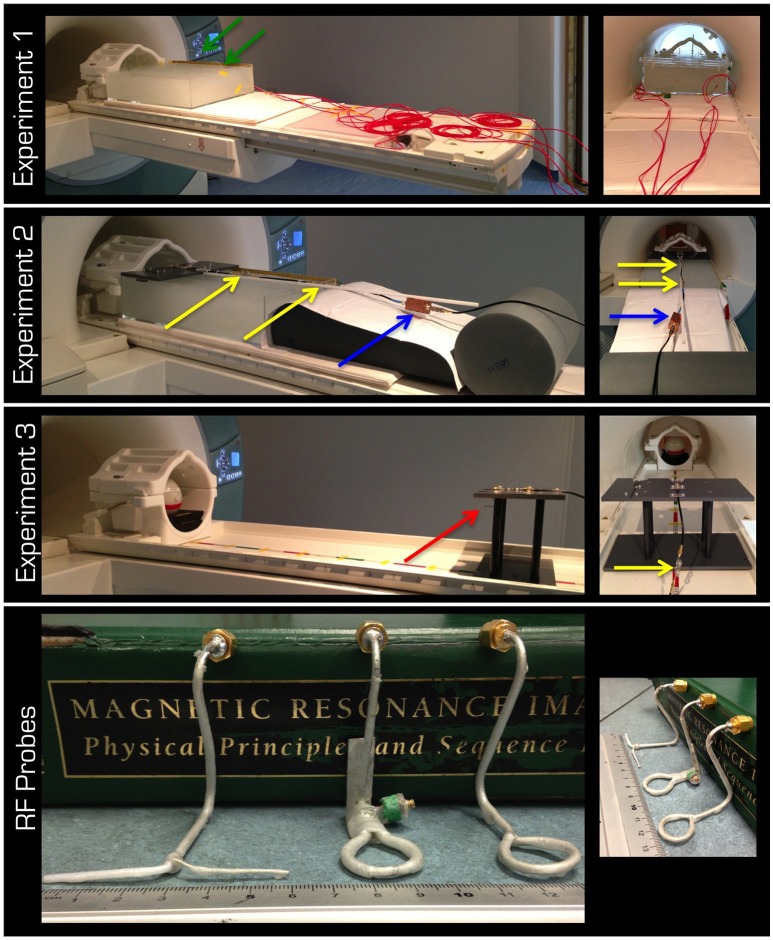
**The top three rows display the experimental set up for Experiments 1–3, respectively**. The lid in Experiment 2 was designed to form the top of the platform in Experiment 3. The red arrow is pointing to the probe in the experiment in air. Note the cable traps (yellow arrows) and the tuning/matching box (blue arrow). The red cabling in the 1st row is the protective cover for the fiber optic connections (green arrows) from the temperature sensors to the signal conditioner, which was kept outside the magnet room. The bottom row displays left to right the non-resonant dipole (**E** field) as well as the resonant and non-resonant loops (**B** field).

All experiments were performed on a 3T Tim Trio scanner (Siemens Healthcare, Erlangen, Germany) using either the Tx/Rx body coil or the Tx/Rx head coil of the vendor (part name: TX/RX CP Head Coil 3T). We performed three experiments (Figure 1). To avoid any gradient-related heating of the phantom inside the bore, a custom-made MRI sequence was implemented in the pulse programming environment of the vendor (IDEA VB17). This sequence transmitted a train of RF rectangular (hard) pulses without imaging gradients. The flip angle and TR were varied to modulate the time-averaged RF power.

**Figure 2:**
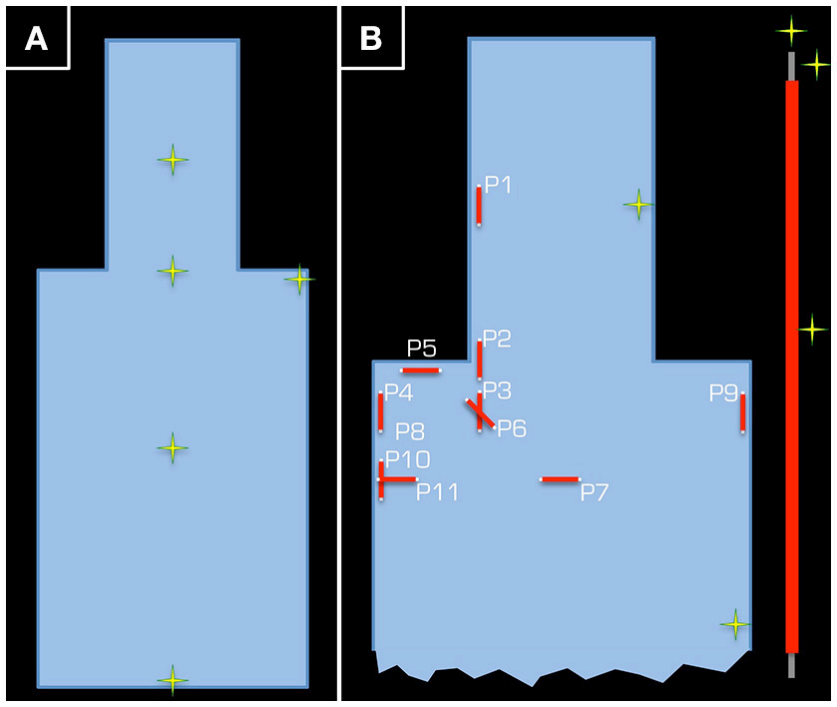
**Positions (yellow/green crosses) in the ASTM phantom at which temperature measurements were taken**. In part **(A)**, the position of the five temperature sensors is given for the control experiment using the Tx/Rx head coil. Each temperature sensor was positioned approximately half way down the depth of the tank. Part **(B)** depicts the set-up of Experiment 1, where two temperature sensors were used as control (one in the head and another in the body of the ASTM phantom), while the other three were kept around the 15-cm long wire (see inset on the right).

The aim of Experiment 1 (Figure 1) was to take temperature measurements in a gel phantom using either the Tx/Rx body or head coil. The gel and phantom were prepared in accordance with the F2182-02a ASTM standard (www.astm.org)^1^, except the depth of the phantom was 15 cm. This depth allowed for the phantom to cover the vertical center of the Tx/Rx head coil (i.e., isocenter of the magnet). The temperature was measured by five optical temperature sensors and a signal conditioner (Opsens, Quebec, Canada) at 100 Hz sampling rate. The acquired data were subsequently low-pass filtered using a moving average with 2 s window. In a control experiment, the five sensors were positioned halfway down the depth of the gel and in a line from the center of the head to the abdomen of the ASTM phantom except one sensor, which was in the left shoulder (Figure 2A). Using the Tx/Rx head coil and registering a human subject with 60 kg body weight the sequence providing 100% SAR was run for 15 min.

In the actual experiment, temperature around a wire that was 15 cm in length was measured. The wire was insulated using PVC along its length, except at the ends, and was positioned in various orientations and positions inside the gel phantom. Three temperature sensors were positioned around the wire. For reference, one channel was left at the side of the head and another at the side of the body of the ASTM phantom (Figure 2B). To maximize the transmitted RF power, the maximum possible body weight of 130 kg (Table 1), under normal operating conditions with regards to the SAR model of the vendor (i.e., although some scanners have options, which allow the user to switch to more lenient safety modes, we performed all experiments in the standard operating mode).

**Table 1:** **Time-averaged RF power vs. body weight for 100% Head SAR while using the body coil**.

**Patient weight (kg)**	**Time-averaged RF power (W)**
50	43.4
60	47.2
70	49.4
80	51.5
90	53.0
100	54.1
110	55.7
120	57.1
130	58.3

RF pulse length, flip angle, and TR were adjusted until a 100% SAR was achieved. It is essential to point out the importance of keeping the position of ASTM phantom on the scanner patient table constant. Regardless of whether the head or body coil was used for transmission, the center of the head part of the ASTM phantom was positioned at the isocenter of the magnet. Such careful positioning is necessary to standardize the measurement, otherwise the scanner may not have adequate information to determine which body part is being scanned. Erroneous guesses of the body part would bias the comparison because SAR models for different body parts are not identical. For the body coil at 100% Head SAR, the time-averaged RF power was 58.2 W. Because these experiments took several days the patient table had to be pulled out and repositioned twice. The time-averaged RF power was 58.4 and 58.3 W for the other occasions. This demonstrates the reproducibility of the experiments. Further aspects of reproducibility and the difficulty in applying standard statistical methods are elaborated in the Appendix. For the experiments with the head coil at 100% Head SAR, the time-averaged RF power was 22.7 W.

Measurements were taken using the Tx/Rx body coil for RF transmit at 11 distinct positions:

1. Side of the head and midway through the axial length of the ASTM phantom head
2. Side of the neck at the junction of the head and body of the ASTM phantom
3. Further toward the body relative to position 2 so that the caudal end of the wire was 30.5 cm away from the isocenter of the magnet
4. Same as position 3 except at the right side of the phantom
5. Wire in the left-right orientation just below the right shoulder
6. Same as position 3 but at an oblique angle in the left-right/head-foot plane
7. Wire in the left-right orientation at the sternum but one end touched the bottom of the phantom
8. Same as position 4 except at the bottom of the phantom
9. Same as position 8 except at the left side of the phantom
10. Wire at the right side of the phantom but 7.5 cm more caudal than position 4
11. Same as position 10 except the wire in the left-right orientation

Positions 1 and 4 were repeated whilst using the Tx/Rx head coil for RF transmission.

In Experiment 2 (Figure 1), the electric (**E**) and the magnetic (**B**) fields transmitted by the Tx/Rx head coil through gel were investigated. Three RF probes (Figure 1) were used to take measurements at distances of up to 60 cm from the coil (i.e., inside the ASTM phantom). The resonant **B** field loop was tuned and matched to at least −25 dB. A partial lid was placed on the ASTM phantom to which the probes could be secured either at the horizontal center (i.e., on-center) or at the horizontal edge (i.e., off-center) of the Tx/Rx head coil. The probes were positioned so that the plane of the loop would be parallel to the surface of the patient table or the dipole would point in the left-right direction. The fitted probes were ~1−2 cm below the gel surface and at the vertical center of the Tx/Rx coil. All probes were connected to the magnet room filter plate via coaxial cables fitted with three appropriate cable traps (>20 dB attenuation at 123.2 MHz) 25 cm apart. The instantaneous pulse RMS voltage induced in each probe was measured with an oscilloscope (Wavelet 300A, Teledyne Lecroy, USA) set to either 50 Ω or 1 MΩ input impedance.

In Experiment 3 (Figure 1), measurements were taken similarly to Experiment 2, but in air. The partial lid from Experiment 2 was designed to form the top of a platform. Measurements of the **E** and **B** fields were taken at distances of up to 105 cm from the coil. Because a spherical gel phantom (Friedman and Glover, 2006) was used for coil loading, no measurement could be taken at the center of the Tx/Rx coil.

Using a commercial finite-difference time domain (FDTD; Yee, 1966) solver (XFdtd, Remcom, State College, PA, USA), we performed simulations for all the above three experiments. The head coil was simulated as a 16-rung coil, driven in the CP1+ mode using current sources with phase shifts in the legs. The body coil was simulated as a 32-rung shielded high-pass birdcage coil following that of Wu et al. (2015). For simulating the **E** and **B** field distributions either a model of the ASTM phantom (as in Experiments 1–2) or a model of a spherical gel phantom (as in Experiment 3) was used.

## Results

**Figure 3:**
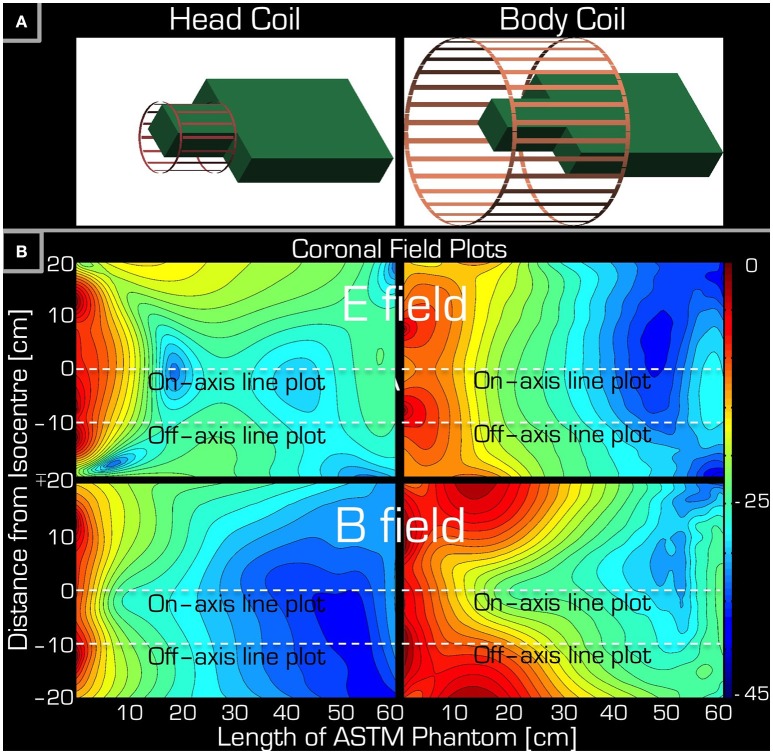
**Simulations accompanying the set-up of Experiment 1**. Part **(A)** depicts the arrangement of the ASTM phantom relative to the Tx/Rx head (left) and Tx/Rx body (right) coils. Part **(B)** shows a coronal section of the ASTM phantom giving both the **E** field (top) and the **B** field (bottom) for both the Tx/Rx head (left) and Tx/Rx body (right) coils. The dashed lines are for reference only (see Figure 5) indicating the left/right center and edge of the Tx/Rx head coil. Please note that each field plot is normalized to its own maximum, hence the colors are not comparable for the four plots.

In the control experiment (i.e., without the conductive wire) the temperature differences between the beginning and the end of the experiment were between −0.2 and 0.1°C. The results of Experiment 1 are listed in Table 2. Using the body coil for RF transmission resulted in excessive heating around the tip of the wire at positions 1 (head), 2 (neck), 4 (upper arm), 5 (shoulder), 6 (upper chest), and 8 (upper arm deep in the tank). Apart from positions 1 and 2, the wire was entirely outside the imaging volume of interest. When using the Tx/Rx head coil for RF transmission, having the wire at position 1 (head) also induced excessive heating. However, in position 4 (upper arm) no heating was measured. The corresponding simulations (Figure 3) are in line with the experimental results in that the upper arm area of the ASTM phantom receives higher **E** field with the Tx/Rx body coil than with the Tx/Rx head coil.

**Table 2:** **Sensors 1 and 2 were positioned at the tip of the wire. Sensor 3 was positioned at the midway point of the wire. Sensor 4 was used as control and positioned next to the inner side of the phantom midway through the body of the ASTM phantom, while sensor 5 (also a control) was positioned at the inner side of the phantom midway through the head of the ASTM phantom (see Figure 2B)**.

	**Sensor 1 (wire tip)**			**Sensor 2 (wire tip)**			**Sensor 3 (wire center)**			**Sensor 4 (control in body)**			**Sensor 5 (control in head)**		
**Pos**	**Begin**	**End**	**Diff**	**Begin**	**End**	**Diff**	**Begin**	**End**	**Diff**	**Begin**	**End**	**Diff**	**Begin**	**End**	**Diff**
**Tx/Rx BODY COIL**															
01	17.8	26.1	8.3	18.3	26.9	8.6	18.2	19.0	0.8	18.1	18.1	0.0	18.5	19.2	0.7
02	16.9	21.3	4.4	17.4	21.7	4.3	18.3	19.0	0.7	18.1	18.1	0.0	19.1	19.8	0.7
03^*^	17.0	16.9	−0.1	17.4	17.3	−0.1	17.8	17.7	−0.1	18.1	18.1	0.0	19.7	19.9	0.2
04	17.6	18.8	1.2	18.1	19.3	1.2	18.5	18.8	0.3	18.2	18.2	0.0	19.8	20.5	0.7
05	18.2	20.0	1.8	18.7	20.5	1.8	18.6	18.9	0.3	18.2	18.2	0.0	20.4	21.0	0.6
06	17.2	18.6	1.4	17.5	18.7	1.3	18.9	19.3	0.4	18.4	18.4	0.0	20.9	21.4	0.5
07	17.0	17.8	0.8	17.5	18.3	0.8	18.0	18.1	0.1	18.3	18.4	0.1	21.3	22.0	0.7
08	18.6	20.4	1.8	19.0	20.6	1.6	18.6	19.0	0.4	18.7	18.6	−0.1	19.6	20.3	0.7
09	18.5	19.2	0.7	18.9	19.5	0.6	18.6	18.7	0.1	18.5	18.5	0.0	20.2	20.8	0.6
10	18.5	19.0	0.5	18.9	19.3	0.4	18.3	18.4	0.1	18.4	18.4	0.0	20.7	21.3	0.6
11	19.7	19.6	−0.1	20.1	20.0	−0.1	19.2	19.3	0.1	18.4	18.4	0.0	21.4	21.9	0.5
**Tx/Rx HEAD COIL**															
01	21.1	23.2	2.1	21.3	25.3	4.0	21.1	22.0	0.8	18.3	18.3	0.0	22.0	22.3	0.3
04	19.3	19.2	−0.1	19.6	19.5	−0.1	18.9	18.8	−0.1	18.4	18.3	−0.1	21.7	22.2	0.5
**REPEATED EXPERIMENTS WITH Tx/Rx BODY COIL FOR QUALITY ASSURANCE**															
01	21.4	25.8	4.4	21.6	30.4	8.8	21.8	23.3	0.5	18.3	18.3	0.0	22.0	22.5	0.5
08	19.2	20.9	1.7	19.6	20.9	1.3	18.9	19.2	0.3	18.3	18.3	0.0	20.8	21.4	0.6

* *For the 3rd position the experiment was stopped after a few minutes because no significant heating was observed*.

**Figure 4:**
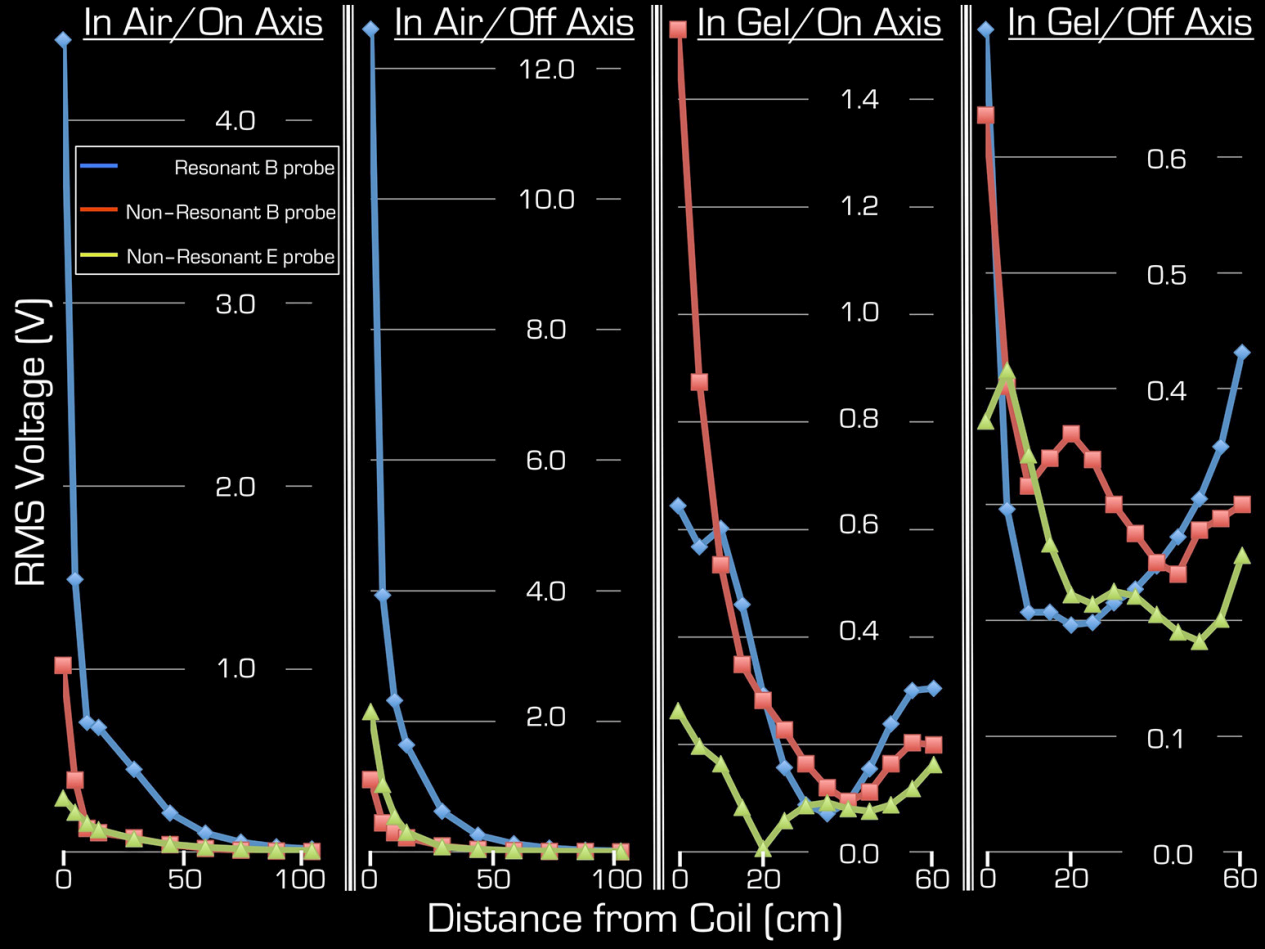
**Both on-axis and off-axis results are shown for Experiment 2 (two right columns) and Experiment 3 (two left columns)**. Note that the y-range is unique to each plot and that the x-range is 60 cm for Experiment 1, whereas for Experiment 2 it is 105 cm. In each plot the blue rhombi, the red squares, and the green triangles represent measurements with the resonant **B** probe, non-resonant **B** probe, and the non-resonant E probe, respectively.

Figure 4 depicts the results of Experiments 2 and 3. As expected, Experiment 3 showed that, in air, the transmitted RF power by the Tx/Rx head coil monotonically decreased with distance both at the center and edge of the coil (1st and 2nd column from the left). Significant power could be detected up to 50 cm from the coil. Use of the resonant **B** field probe resulted in several fold higher measured RMS power than use of the non-resonant **E** or **B** field probes. Closer to the edge of the coil, in the vicinity of the electronic components of the coil, the measured power of the **E** field was higher than that of the **B** field.

Using the gel phantom, measurements in Experiment 2 produced a more complex behavior (3rd and 4th columns from the left). Rather than a monotonic decrease with distance, several local extrema were observed, and at the end of the ASTM gel phantom (farthest from the Tx/Rx head coil) local maxima were apparent with all three probes used in this experiment. For example, at the foot end of the phantom (60 cm away from the birdcage coil), the on-axis measurements of the **E** and **B** field magnitudes exceeded 48, 13, and 63% for the resonant**/**non-resonant **B** probes and the **E** probe, respectively, when compared to the corresponding measurements at the coil (i.e., 0 cm away). In particular, the resonant B probe produced higher/lower results than the non-resonant probe, depending on spatial position.

Because the ASTM phantom had a head segment, the spherical gel phantom was no longer needed for loading the Tx/Rx coil. This allowed for an extra measurement at the center of the Tx/Rx head coil. As expected, measurements with all three probes provided the highest results at this position: 4.25/4.9/1.7 V (RMS) for the resonant/non-resonant **B** field probes and the non-resonant **E** field probe, respectively.

**Figure 5:**
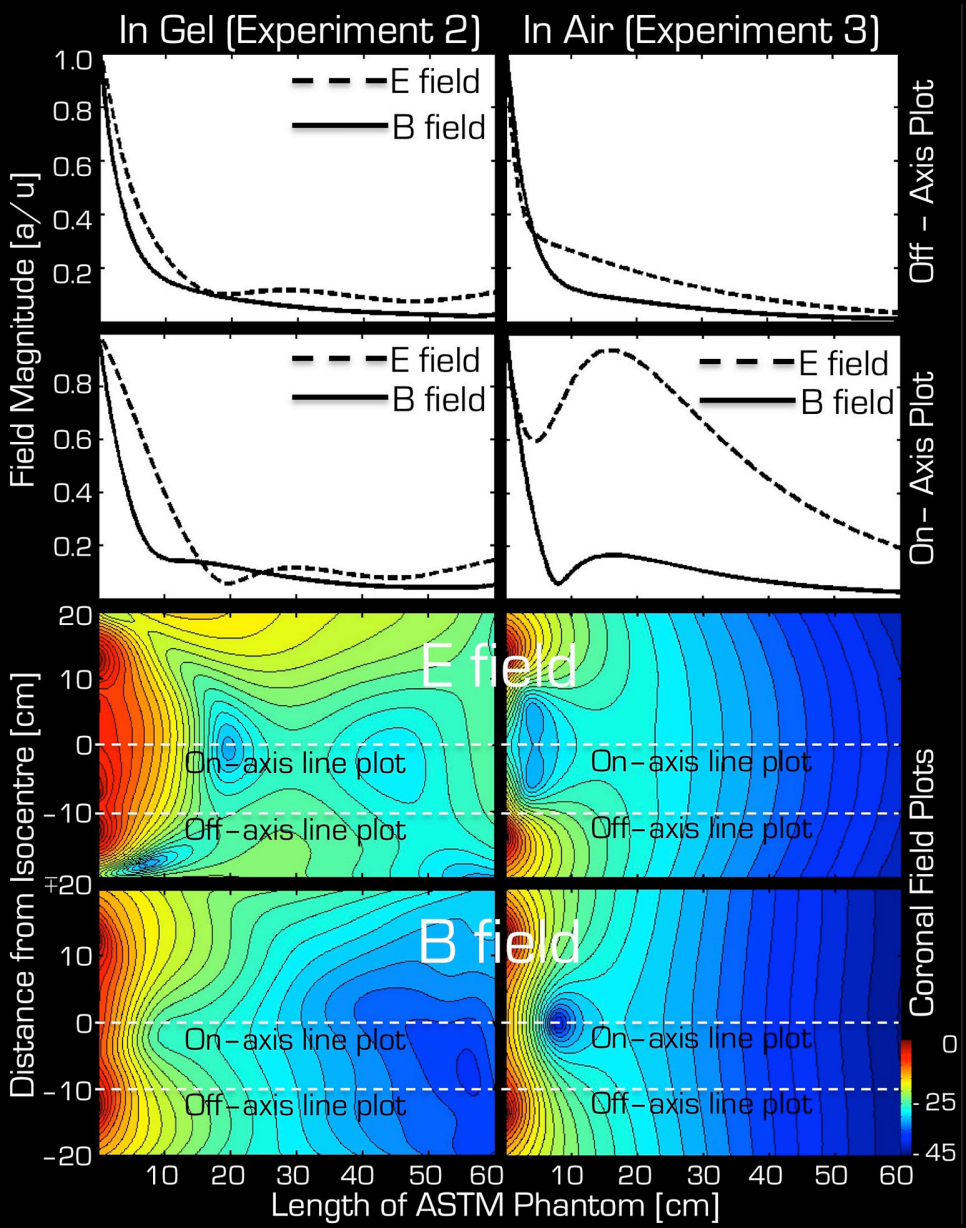
**Simulation results for Experiment 2 (left) and Experiment 3 (right)**. The top two rows depict the profile of simulated field intensity along a straight line down the center (on-axis) or around edge (off-axis) of the Tx/Rx head coil (see dashed white lines in corresponding two field plots on the bottom). On the bottom, simulated coronal slices through the ASTM phantom are shown for the E (3rd row) and B (bottom row) fields. The color bar represents attenuation (in dB) relative to the maximum value (red or 0 dB).

The simulations were in qualitative agreement with the experimental results (Figure 5). There was an almost entirely monotonic decrease of the transmitted **E** and **B** fields in the simulations of Experiment 3 in air (right column). The complex behavior of Experiment 2 in the ASTM phantom was also confirmed by the simulation results (left column). In particular, the surprising effect of the local maxima at the end of the ASTM phantom was clearly apparent in the simulations. For the in-air case and in the vicinity of the coil, the simulations and experimental results (Figure 4) supported each other well in that both the **E** and resonant **B** fields were higher near the rungs (off-axis) than at the center (on-axis) of the Tx/Rx coil when considering the resonant **B** and the **E** field probes. For the case of the ASTM phantom, the simulations also indicated that the **B** field magnitude would be slightly lower on axis than off axis. Experiments with the resonant **B** field probe confirmed this prediction. For the case of the ASTM gel phantom, the simulations differed slightly from the experimental results for the **E** field. The simulations predicted similar E field magnitude on and off axis in the vicinity of the coil. Experimentally, we found that the **E** field magnitude was higher off axis than on axis.

## Discussion

We have shown that conductors can heat up significantly in response to RF transmit fields even if they are entirely outside the imaging volume of interest. In particular, during a neuro examination the 15 cm long wire was a risk for both the Tx/Rx body coil and the Tx/Rx head coil when in the shoulder region. However, in the upper arm region the Tx/Rx head coil caused no noticeable heating, while in the same position the Tx/Rx body coil did in fact cause unacceptable heating. Together with the simulations and experiments in both gel and air, these results show that at 3T the Tx field of the Tx/Rx birdcage head coil extends well-beyond its physical dimensions. In particular, when a conductive medium, such as a human, is inside or near coil the transmitted **E** and **B** fields display a more complex behavior than a simple monotonic decay with distance.

Although transmitted **E** and **B** fields have been studied for a Tx/Rx body coil (Amjad et al., 2005), little is known about the safety of the Tx/Rx head coil configuration in cases where an implant is located entirely outside of the volume of the Tx/Rx head coil. Most incidence reports concern cases where at least part of the implant is within the imaging volume. However, a recent report also investigated whether conductive implants posed a risk when placed in an area of the body that was outside the imaging volume of interest (Noureddine et al., 2015). Their investigations only concerned measurements at 7T, but concluded that a categorical exclusion of volunteers with implants is overly conservative.

It should be noted that *ex vivo* experiments, which investigate induced RF heating in or around implants, could have different outcomes depending on the phantom composition. Specifically, performing the experiment in a saline solution produces much less heating than that in a gelled agent (Park et al., 2003). The phantom used in this study conformed to the ASTM standard (F2182-02a, www.astm.org), which requires a gelled phantom material. Therefore, our results are unlikely to be an underestimation.

The amount of heating around a conductive wire depends on a combination of several parameters, including, among other things, length, thickness of its insulation, and conductance of the surrounding medium. For example, Yeung et al. (2002) found that wires that were insulated, except at their tips, produced up to 10 times more heating than those that were completely insulated. The straight conductor used in this experiment was designed this way in order to exacerbate potential RF-induced heating.

To increase the sensitivity of our measurement to the potential heating in Experiment 1, we positioned three temperature sensors around the conductor. The manufacturer advised to position these sensors (crystal strain gauges) both parallel to the tip of the conductor and perpendicular to it. The third temperature sensor was positioned at the center of the wire because different mechanisms cause heating at the tip and at the center of the conductor.

The simulations predicted the experiments very well. One deviation occurred in the E field near the coil (neck and shoulder area in a human subject) in the ASTM gel phantom. In this case the simulations predicted that the E field would be similar both on and off axis (i.e., at the center of the coil vs. the rungs) but Experiment 2 indicated that closer to the rungs the **E** field was higher. This discrepancy may be due to the capacitors being near the rungs, which store the E field.

Another noteworthy point is that use of the resonant **B** field probe did not provide the highest measurement of RMS power on axis in the gel phantom in Experiment 2. This may be due to the large number of variables, which are hard to control for simultaneously. We used a network analyzer to tune and match the resonant probe separately for each of the experimental set ups. While for all experiments the matching was at least −25 dB, at different positions the tuning/matching was variable.

It is interesting that both the simulations and the experimental results indicate that the RF induced **E** and **B** fields show local maxima at the end of the ASTM phantom (farthest from the Tx/Rx coil) as opposed to a monotonic decrease as one might expect from the in air experiments. These local maxima may be due to a skin effect at the caudal edge of the phantom. An able-bodied participant or clinical patient would have their lower extremities at this point. Nevertheless, these results are noteworthy for implants that are far from the Tx/Rx head coil but superficially placed within the body.

There were limitations to this study. Generally, the simulations describe an idealized version of the real experiments. Perhaps the most marked difference is the size of the probes. In the experiments the probes have a finite size and hence can therefore interact with the Tx coil, perturbing its field. On the other hand, in the simulation a point conductor is assumed without such interaction. Because in these experiments the probes were electrically small, this point source approximation is reasonable for the non-resonant probes. A refinement for future studies would be to model a resonant loop and to run multiple simulations at different positions to investigate how perturbed the Tx field is. It would also be possible to make very small (5 mm diameter/length) **E** and **B** field probes and thus approximate the simulated point measurements more closely. These point measurements could be made on a Cartesian grid spanning all three axes, similar to the measurements performed by Nordbeck et al. (2008) with a body coil. Further, it must be noted that relying solely on SAR-values as reported by the scanner is generally not recommended (Baker et al., 2004) because the implementation is manufacturer specific and hence it provides an unreliable comparison. We would like to point out that in the present paper the same scanner was used for all experiments, and careful placement of the phantom on the patient table ensured that the same SAR model and the same time-averaged RF power were used.

It is important to note that many elements of MRI scanners are manufacturer specific. For example, the length of the Tx/Rx body coil, the length of the bore (i.e., the size of the magnet), and the diameter of the bore are often obvious. A less obvious factor that may contribute to heating and resonance effects is the actual field strength of the scanner. Different manufacturers ramp their magnets to slightly different fields. Also, when several scanners are installed nearby they are often ramped to slightly different field strengths to avoid cross talk. This is not an exhaustive list of engineering issues that could modulate the amount of heating around conductive implants but it demonstrates the difficulties in generalizing such results and highlights the caution necessary when interpreting reports on heating from the literature. By testing resonant probes and insulated wires, the experiments were designed to reflect worst-case, or at least high-risk, scenarios with respect to the implant. However, it is still possible that other configurations may pose an even higher risk. Thus, we recommend caution when extrapolating to other cases and configurations.

## Conclusion

At 3T, our findings support the use of the Tx/Rx head coil in favor of the body coil for examinations involving individuals with abdominal or lower thoracic implants. Although we have shown that the induced E and B fields of the Tx/Rx head coil extend further out than its physical dimensions, in our experiments at 3T we could not detect any heating related to these fields while using the head coil. One must still exercise caution since our study may not have included a worst-case scenario, which is difficult to predict and achieve experimentally (Kainz, 2007). Implants close to the neck and shoulders would especially need to be considered very carefully.

## Author contributions

ZN, AO-T, and NW designed the study, performed the experiments, and analyzed the data. AK and SG performed the simulations. ZN drafted the manuscript. The submitted version is the combined effort of all authors.

## Funding

This work and open access of the article were supported by the Wellcome Trust (WT 091593/Z/10/Z).

### Conflict of interest statement

The authors declare that the research was conducted in the absence of any commercial or financial relationships that could be construed as a potential conflict of interest.

## Footnotes

^1^ASTM Standard F2182-02a.

## Appendix

Although the present article seems devoid of standard statistical analysis, it is not an oversight. Statistical analyses are based on the assumption that measurements are made under identical conditions. Hence, the results of these repeated experiments are expected to be reproducible apart from random errors. However, the ASTM standard on RF-induced heating (F2182-02a, www.astm.org) does not describe repeated measurements. Instead it requires 15 min of measurement with and without the implant (in our case the straight wire) using the same scanner hardware and MR sequence. The measurement without the implant serves as a control. We implemented two controls. The first is the separate control heating experiment (Figure 2A) without the wire in place. Secondly, in each experiment that involved the straight wire in Experiment 1 (Figure 2B), we used two additional temperature sensors (one in the head and another in the body of the ASTM phantom). These sensors were left in the same position for all experiments to monitor a baseline (either heating by the RF transmission or the air conditioning of the room). Table 2 indicates that although the actual temperature of the phantom changed over the experiments, the change in temperature during the 15 min of heating tests were highly stable (see sensors 4 and 5). The Opsens temperature sensors are capable of 1 kHz sampling rate. We recorded our data at 100 Hz. The actual values that are compiled in Table 2 can be considered highly precise, given that they represent the average of at least 30 s of measurement (i.e., 30000 samples or 3000 recorded data points) right before and at the end of the 15 min heating period.

The heating measurements presented in this paper are extremely difficult and time consuming. Small changes in the position or orientation of the conductive object can provide drastically different results. Furthermore, the heating produced is often very local. Therefore, the results are also very sensitive to small changes in the position of the temperature sensors relative to the conductive object. Nonetheless, even if repeated measurements were made, say 10 times in each of the 10 positions described in the methods, standard statistical analyses would usually not be performed. Instead the measurement, which produced the highest amount of heating at each of the 10 positions would be taken. This would represent the worst-case scenario, which is of paramount interest in safety measurements. Nonetheless, temperature measurements were repeated in two positions whilst using the body coil for RF transmission (Table 2). Rather than leaving the wire in the same position and simply allowing the gel to cool down, the repeated measurement was taken far apart in time so that the wire, the wire holder, the temperature sensors (sensors 1–3) had to be repositioned fully.

To avoid some sources of systematic errors (i.e., those that depend on time), the measurements in Experiment 2–3 were taken in a random order. Furthermore, some measurements, at different distances, were repeated for quality assurance. These repeated measurements were also taken randomly in time. For example, in Experiment 2 we had 94 vs. 93 mV at 15 cm, 62 vs. 66 mV at 25 cm, 91 vs. 96 mV at 40 cm using the **E** field dipole for the on-axis measurements; 2460 vs. 2530 mV at 0 cm and 282 vs. 300 mV at 30 cm using the non-resonant **B** field probe for the on-axis measurement. For all but one of the 18 repeated measurements the discrepancy was within 6%. The measurement with a 13% discrepancy was taken in air, off-axis, at a distance of 105 cm from the coil, and using the non-resonant **B** field probe. The two measurements were 4.65 vs. 5.3 mV.

As mentioned in the Materials and Methods Section, positioning the phantom was also highly reproducible. The three times that positioning was necessary the time-averaged RF transmit power was within 0.2 W (~0.3%).

## References

[B1] Amjad A. Kamondetdacha R. Kildishev A. V. Park S. M. Nyenhuis J. A. (2005). Power deposition inside a phantom for testing of MRI heating. IEEE Trans. Magn. 41, 4185–4187. 10.1109/TMAG.2005.854840

[B2] Armenean C. Armenean M. Perrin J. (2004a). RF heating comparison between conductive and resistive wires in interventional and endoluminal MRI, in Proceedings of the 12th Annual Meeting of ISMRM (Kyoto), 667.

[B3] Armenean C. Perrin E. Armenean M. Beuf O. Pilleul F. Saint-Jalmes H. (2004b). RF-induced temperature elevation along metallic wires in clinical magnetic resonance imaging: influence of diameter and length. Magn. Reson. Med. 52, 1200–1206. 10.1002/mrm.20246 15508156

[B4] Baker K. B. Tkach J. A. Nyenhuis J. A. Phillips M. Shellock F. G. Gonzalez-Martinez J.. (2004). Evaluation of specific absorption rate as a dosimeter of MRI-related implant heating. J. Magn. Reson. Imaging 20, 315–320. 10.1002/jmri.20103 15269959

[B5] Benbadis S. R. Nyhenhuis J. Tatum W. O. Murtagh F. R. Gieron M. Vale F. L. (2001). MRI of the brain is safe in patients implanted with the vagus nerve stimulator. Seizure 10, 512–515. 10.1053/seiz.2001.0540 11749109

[B6] Carmichael D. W. Pinto S. Limousin-Dowsey P. Thobois S. Allen P. J. Lemieux L.. (2007). Functional MRI with active, fully implanted, deep brain stimulation systems: safety and experimental confounds. Neuroimage 37, 508–517. 10.1016/j.neuroimage.2007.04.058 17590355

[B7] Dempsey M. F. Condon B. Hadley D. M. (2001). Investigation of the factors responsible for burns during MRI. J. Magn. Reson. Imaging 13, 627–631. 10.1002/jmri.1088 11276109

[B8] Friedman L. Glover G. H. (2006). Report on a multicenter fMRI quality assurance protocol. J. Magn. Reson. Imaging 23, 827–839. 10.1002/jmri.20583 16649196

[B9] Henderson J. M. Tkach J. Phillips M. Baker K. Shellock F. G. Rezai A. R. (2005). Permanent neurological deficit related to magnetic resonance imaging in a patient with implanted deep brain stimulation electrodes for Parkinson's disease: case report. Neurosurgery 57, E1063. discussion: E1063. 10.1227/01.NEU.0000180810.16964.3E 16284543

[B10] Kainz W. (2007). MR heating tests of MR critical implants. J. Magn. Reson. Imaging 26, 450–451. 10.1002/jmri.21020 17729357

[B11] Kanal E. Barkovich A. J. Bell C. Borgstede J. P. Bradley W. G. Froelich J. W.. (2013). ACR guidance document on MR safe practices: 2013. J. Magn. Reson. Imaging 37, 501–530. 10.1002/jmri.24011 23345200

[B12] Konings M. K. Bartels L. W. Smits H. F. Bakker C. J. (2000). Heating around intravascular guidewires by resonating RF waves. J. Magn. Reson. Imaging 12, 79–85. 10.1002/1522-2586(200007)12:1<79::AID-JMRI9>3.0.CO;2-T 10931567

[B13] Luechinger R. Zeijlemaker V. A. Pedersen E. M. Mortensen P. Falk E. Duru F.. (2005). *In vivo* heating of pacemaker leads during magnetic resonance imaging. Eur. Heart J. 26, 376–383. discussion: 325–327. 10.1093/eurheartj/ehi009 15618060

[B14] Mattei E. Triventi M. Calcagnini G. Censi F. Kainz W. Mendoza G.. (2008). Complexity of MRI induced heating on metallic leads: experimental measurements of 374 configurations. Biomed. Eng. Online 7:11. 10.1186/1475-925X-7-11 18315869 PMC2292730

[B15] Nitz W. R. Oppelt A. Renz W. Manke C. Lenhart M. Link J. (2001). On the heating of linear conductive structures as guide wires and catheters in interventional MRI. J. Magn. Reson. Imaging 13, 105–114. 10.1002/1522-2586(200101)13:1<105::AID-JMRI1016>3.0.CO;2-0 11169811

[B16] Nordbeck P. Fidler F. Weiss I. Warmuth M. Friedrich M. T. Ehses P.. (2008). Spatial distribution of RF-induced E-fields and implant heating in MRI. Magn. Reson. Med. 60, 312–319. 10.1002/mrm.21475 18666101

[B17] Noureddine Y. Bitz A. K. Ladd M. E. Thürling M. Ladd S. C. Schaefers G.. (2015). Experience with magnetic resonance imaging of human subjects with passive implants and tattoos at 7 T: a retrospective study. MAGMA 28, 577–590. 10.1007/s10334-015-0499-y 26410044

[B18] Nyenhuis J. A. Bourland J. D. Foster K. S. Graber G. P. Terry R. S. Adkins R. A. (1997). Testing of MRI compatibility of the cyberonics model 100 NCP generator and model 300 series lead. Epilepsia 38, 140

[B19] Nyenhuis J. A. Park S.-M. Kamondetdacha R. Amjad A. Shellock F. G. Rezai A. R. (2005). MRI and implanted medical devices: basic interactions with an emphasis on heating. IEEE Trans. Device Mater. Reliability 5, 467–480. 10.1109/TDMR.2005.859033

[B20] Park S. M. Nyenhuis J. A. Smith C. D. Lim E. J. Foster K. S. Baker K. B. (2003). Gelled versus nongelled phantom material for measurement of MRI-induced temperature increases with bioimplants. IEEE Trans. Magnet. 39, 3367–3371. 10.1109/TMAG.2003.816259

[B21] Purcell E. M. Morin D. J. (2013). Electricity and Magnetism. Cambridge: Cambridge University Press.

[B22] Rezai A. R. Phillips M. Baker K. B. Sharan A. D. Nyenhuis J. Tkach J.. (2004). Neurostimulation system used for deep brain stimulation (DBS): MR safety issues and implications of failing to follow safety recommendations. Invest. Radiol. 39, 300–303. 10.1097/01.rli.0000124940.02340.ab 15087724

[B23] Sankar T. Lozano A. M. (2011). Magnetic resonance imaging and deep brain stimulation: questions of safety. World Neurosurg. 76, 71–73. 10.1016/j.wneu.2011.04.013 21699483

[B24] Shellock G. (2013). Reference Manual for Magnetic Resonance Safety Implants and Devices, 2013 Edn. Philadelphia, PA: Biomedical Research Publishing Group.

[B25] Shellock G. Kanal E. (1996). Magnetic Resonance: Bioeffects, Safety and Patient Management. Los Angeles, CA: Lippincott Williams & Wilkins.

[B26] Smith C. D. Kildishev A. V. Nyenhuis J. A. Foster K. S. Bourland J. D. (2000). Interactions of magnetic resonance imaging radio frequency magnetic fields with elongated medical implants. J. Appl. Phys. 87, 6188–6190. 10.1063/1.372651

[B27] Spiegel J. Fuss G. Backens M. Reith W. Magnus T. Becker G.. (2003). Transient dystonia following magnetic resonance imaging in a patient with deep brain stimulation electrodes for the treatment of Parkinson disease. Case report. J. Neurosurg. 99, 772–774. 10.3171/jns.2003.99.4.0772 14567615

[B28] Wu X. Zhang X. Tian J. Schmitter S. Hanna B. Strupp J.. (2015). Comparison of RF body coils for MRI at 3 T: a simulation study using parallel transmission on various anatomical targets. NMR Biomed. 28, 1332–1344. 10.1002/nbm.3378 26332290 PMC4573930

[B29] Yee K. (1966). Numerical solution of initial boundary value problems involving maxwell's equations in isotropic media. IEEE Trans. Antennas Propagation 14, 302–307. 10.1109/TAP.1966.1138693

[B30] Yeung C. J. Susil R. C. Atalar E. (2002). RF safety of wires in interventional MRI: using a safety index. Magn. Reson. Med. 47, 187–193. 10.1002/mrm.10037 11754458

[B31] Zrinzo L. Yoshida F. Hariz M. I. Thornton J. Foltynie T. Yousry T. A.. (2011). Clinical safety of brain magnetic resonance imaging with implanted deep brain stimulation hardware: large case series and review of the literature. World Neurosurg. 76, 164–172. discussion: 69–73. 10.1016/j.wneu.2011.02.029 21839969

